# Influence of Environmental Factors on Injury Severity Using Ordered Logit Regression Model in Limpopo Province, South Africa

**DOI:** 10.1155/2022/5040435

**Published:** 2022-02-21

**Authors:** Peter M. Mphekgwana

**Affiliations:** Research Administration and Development, University of Limpopo, Polokwane, South Africa

## Abstract

Globally, road traffic accidents are a major cause of death and severe injuries. It is estimated that the number of deaths on the world's roads at 1.5 million per annum puts road traffic injuries as the eighth leading cause of death globally. Understanding the influence of environmental factors on deaths and severe injuries will help in policy-making and the development of strategies in Limpopo Province. We, therefore, aim to study environmental factors that influence road deaths and severe injuries and to identify whether their impact on injury severity levels varies. The study was based on secondary data on road traffic accidents obtained from the Department of Roads and Transport in Limpopo Province. The data comprised 18 029 road traffic accidents for the period January 2009–December 2015. The study found that weekends (Saturdays and Sundays) had the highest number of accidents when compared to weekdays. The proportion of observations in each severity level was not constant across explanatory variables. The generalized ordered logit regression (GOLR) models seemed to be an effective predicting model that can be adapted to determine the influence of environmental factors on injury severity compared to the ordered logit regression (OLR) model. The results of the GOLR model suggest that environmental factors such as slippery road conditions, rainy weather, and spring season lower the likelihood of severe crash occurrence. On the other hand, poor or defective road surface, time interval (6 a.m. to 11 p.m.), and provincial roads have a higher likelihood of severe crash occurrence. To decrease the severity of injuries in the province, provincial roadways must be maintained regularly.

## 1. Introduction

Globally, Road Traffic Accidents (RTAs) are a major cause of death and severe injuries. In its 2018 report, the World Health Organization (WHO) estimated the number of deaths on the world's roads at 1.5 million per annum and put road traffic injuries as the eighth leading cause of death globally [1]. It also stated that between 20 and 50 million more people suffer nonfatal injuries, with many incurring a disability as a result of their injuries. The social and economic costs of deaths and injuries due to RTAs are over US$100 billion annually [2]. Also, RTAs were seen to be some of the main leading causes of death among people under the age of 40 years [3].

Regionally, Africa, with only 2% of the world's vehicles, is the least motorized region of the world but contributes 16% of the global traffic deaths with Nigeria and South Africa contributing a significant proportion of these deaths [1]. Overall, young Africans aged between 15 and 29 years are most vulnerable to these RTAs [1]. The burden of Road Traffic Deaths (RTDs) is reported to be on the rise in developing countries such as South Africa despite increased efforts to address these traffic accidents. The 2016 report of the Road Traffic Management Corporation states that the number of RTDs increased by 10% between the years 2014 and 2015 [4]. This annual road carnage costs the South African economy approximately R43 billion, of which 60% is due to damage to vehicles and other properties [5, 6].

In 2015, 10 613 fatal accidents occurred between January and December 2015 and resulted in 12 944 fatalities in the country with a rate severity of 1.2 [7]. Previous studies in this field have identified various factors that are associated with RTAs and RTDs. These include, among others, sociodemographics such as the age of the driver, gender, speeding, not wearing a seat belt, vision deficiency that is corrected with the eyeglass, dark but lighted conditions (07:30 pm to 05:30 am), rainy seasons, drivers under the influence of drugs or alcohol, drivers exhibiting aggressive driving behaviour, and negative road engineering factors as well as quality of pavement [8–13].

To the best of our knowledge, information on the influence of environmental factors on death and severity of injuries on Limpopo Province roadway highways is still limited. It is, therefore, in this context that the present study aimed to contribute to the literature on environmental risk factors associated with deaths and severe injuries and to identify whether their impact on injury severity levels varies. In doing so, we use the ordered logit regression model (OLR) and will consider some extensions with the ultimate goal of producing a robust model for modeling death and severity injuries in the province. Researching on the impact of environmental factors on death and severe injuries in Limpopo Province of South Africa is of particular relevance to government and public health in the development of strategies to reduce road deaths and severe injuries.

## 2. Literature Review

A previous study conducted in South Africa by Vogel and Bester classified risk factors influencing road traffic crashes as human, vehicle, and road and environment factors [14]. Human factors included negligence, excess speed, dangerous overtaking, pedestrians in the road, and inconsiderate driving behaviour. Vehicle factors were mostly about faulty brakes and tyres. Road and environmental factors included rush-hour traffic and inadequate facilities for pedestrians. The study found that human factors accounted for 75% of the accidents, followed by environmental (15%) and vehicle factors (10%) [14].

Various studies have shown that environmental factors were significantly related to risk road fatalities. A study conducted by Jung et al. in southeastern Wisconsin assessed the effects of rainfall on the severity of single-vehicle accidents, taking into account weather-related factors such as estimated rainfall intensity for 15 minutes before accident occurrence, water film depth, temperature, wind speed/direction, stopping sight distance, and car-following distance at the time of the crash [15]. The study found that rainfall intensity, wind speed, and horizontal or vertical curves were all linked to an increased likelihood of accident severity in rainy weather. More accidents commonly occurred inroads with two-way traffic as opposed to single-way roads [16]. Furthermore, the risk decreases with the width of the road. Moreover, studies have found a link between road accident frequency and risk factors such as road segment length, width, number of ramps and bridges, horizontal and vertical curves, and shoulder-width [17].

A high proportion of road accidents and deaths occur during the night between 6 p.m. and 6 a.m. and during peak times between 12 p.m. and 6 p.m. [12]. Some accidents are caused by a lack of street lights, particularly during nighttime driving on undivided 2-lane or 2-way rural highways [18]. This could lead to difficulty in distinguishing the lane separation which might cause an accident. The probability of fatality is estimated to rise when dull lighting conditions are present [13]. Multivehicle accidents commonly occur in off-peak hours during the daytime [8].

Modeling of traffic accident injury severity is complex and has received more attention in recent years [19]. Several statistical models have been used to estimate the severity of traffic injuries. A study done in New Mexico from 2010 to 2011 used multinomial logit regression models to investigate the characteristics that differentiate teenage and adult drivers in intersection-related accidents [20]. However, it should be emphasized that the injury severity response variable has the nature of the scale that underlies the items (no injury, minor injury, severe injury, fatality) which renders the multinomial logit regression model unsuitable for analyzing injury severity. A recent study conducted in China investigating the statistical distribution characteristics such as types of environmental properties and road properties deployed an ordered logit regression (OLR) model to account for the unobserved heterogeneity across observations and also cater for choices that have an inherent order to them [21]. OLR models are among the most common ordinal regression techniques; however, they often have serious shortcomings [22]. These approaches frequently violate the proportionate odds/parallel lines assumptions. Model misspecification can cause issues that are worse than the ones that these strategies were designed to address. One of the alternatives to address violated proportional assumption is to use generalized ordered logit regression (GOLR) models as they may emphasize a proportion of observations in each level of the response if not consistent across each level of the explanatory variable [22].

## 3. Materials and Methods

### 3.1. Study Setting, Population, and Data Collection

This study is based on secondary data on RTAs obtained from the Limpopo Provincial Department of Roads and Transport. Limpopo is the northernmost province of South Africa with an estimated population of 5 million. It comprises five districts, namely, Capricorn, Mopani, Sekhukhune, Vhembe, and Waterberg. The data comprised 18 029 RTAs over the period 2009 January–December 2015. A single record for each accident was created along with a set of variables indicating severity, time of the accident, locations, and the cause as shown in Table 1.

### 3.2. Statistical Analysis

All data analyses were performed using SPSS version 26.0 (IBM SPSS Statistics) (IBM, Chicago, USA) and *R* software. Baseline characteristics of the RTAs were expressed as frequencies and percentages, and the Mantel-Haenszel extension test was used to test for linear trends in the injury severity level.

#### 3.2.1. Ordered Logit Regression Model (OLR)

The ordered logit regression model (OLR) was applied to determine the relationship and determinants of death and severe injuries. Suppose *Y* denotes the ordinal outcome with *j* categories and *µ* is the corresponding conditional mean. The odds ratio of being less than or equal to particular *j* categories is given as

(1) $$\begin{array}{c} \frac{P_{r}\left[Y_{i}\leq j|X\right]}{P_{r}\left[Y_{i}>j|X\right]}=\frac{P_{r}\left[Y_{i}\leq j|X\right]}{P_{r}\left(1-P_{r}\left[Y_{i}\leq j|X\right]\right)},\;j=1,2,3,4. \end{array}$$

Considering ordered logit function

(2) $$\begin{array}{c} \text{logit}\left(P_{r}\left[Y_{i}\leq j|X\right]\right)=\text{In}\left(\frac{P_{r}\left[Y_{i}\leq j|X\right]}{P_{r}\left[Y_{i}>j|X\right]}\right)=\text{In}\left(\frac{P_{r}\left[Y_{i}\leq j|X\right]}{P_{r}\left(1-P_{r}\left[Y_{i}\leq j|X\right]\right)}\right),\;j=1,2,3,4. \end{array}$$

The OLR is defined as

(3) $$\begin{array}{c} \text{logit}\left(P_{r}\left[Y_{i}>j\right]\right)=-a_{j}+\beta x_{i}^{\prime },\;\left\{\begin{array}{c} j=1,2,3,4,\\ i=1,\ldots ,n, \end{array}\right. \end{array}$$

where *a* is the slope of the model, *β* is the coefficient of the model, *i* is the explanatory variable, and *j* is the severity level (property damage, minor injuries, serious injuries, and death). Then *β* is assumed to be the same for all the explanatory variables.

#### 3.2.2. Generalized Ordered Logit Regression (GOLR)

The generalized ordered logit regression model (GOLR) was applied to determine the relationship and determinants of death and severe injuries. Suppose *Y* denotes the ordinal outcome with *j* categories and *µ* is the corresponding conditional mean. The odds ratio of being less than or equal to particular *j* categories is given as

(4) $$\begin{array}{c} \frac{P_{r}\left[Y_{i}\leq j|X\right]}{P_{r}\left[Y_{i}\leq j|X\right]}=\frac{P_{r}\left[Y_{i}\leq j|X\right]}{P_{r}\left(1-P_{r}\left[Y_{i}\leq j|X\right]\right)},\;j=1,2,3. \end{array}$$

Considering ordered logit function

(5) $$\begin{array}{c} \log\;it\left(P_{r}\left[Y_{i}\leq j|X\right]\right)=\text{In}\left(\frac{P_{r}\left[Y_{i}\leq j|X\right]}{P_{r}\left[Y_{i}>j|X\right]}\right)=\text{In}\left(\frac{P_{r}\left[Y_{i}\leq j|X\right]}{P_{r}\left(1-P_{r}\left[Y_{i}\leq j|X\right]\right)}\right),\;j=1,2,3,4. \end{array}$$

The GOLR regression model is defined as

(6) $$\begin{array}{c} \text{logit}\left(P_{r}\left[Y_{i}>j\right]\right)=-a_{j}+\sum_{1}^{n}\beta_{i}x_{i},\;\left\{\begin{array}{c} j=1,2,3,4,\\ i=1,\ldots ,n, \end{array}\right. \end{array}$$

where *a* is the slope of the model, *β* is the coefficient of the model, *i* is the explanatory variable, and *j* is the severity level (property damage, minor injuries, serious injuries, and deaths). Then *β* is not the same for all the explanatory variables.

#### 3.2.3. K-Means Clustering

K-means clustering is a popular method for cluster analysis in data mining. It partitions *n* observations into K clusters in which each observation belongs to the cluster with the nearest mean. Given a set of observations (*x*_1_, *x*_2_,…, *x*_*n*_), where each observation is a *d*-dimensional real vector, K means clustering aims to partition the observation into *K*(≤*n*) sets to minimize the within-cluster sum of squares.

#### 3.2.4. Brant Test

The Brant test developed by Rollin Brant in 1990 was used in the study to assess whether the observed deviations from the OLR model are larger than what could be attributed to chance alone [23].

(7) $$\begin{array}{c} \eta_{j}=\left\{\begin{array}{cc} 1, & \text{if,}\;y>j,\\ 0, & \text{if,}\;y\leq j, \end{array}\;j=1,2,3\right., \end{array}$$

with success probability, *π*_*j*_=*P*_*r*_[*η*_*j*_=1]=1 − *γ*_*j*_ satisfy. logit(*π*_*j*_)=−*a*_*j*_+*βx*_*i*_′.

### 3.3. Wald-Type Goodness-of-Fit Statistic

(8) $$\begin{array}{c} \chi^{2}={\left(D\hat{\beta }\right)}^{'}{\left[D\hat{V}\hat{\beta }D^{\prime }\right]}^{-1}\left(D\hat{\beta }\right)∼\chi_{\left(k-2\right)p}^{2}, \end{array}$$

where

(9) $$\begin{array}{c} D=\left[\begin{array}{ccc} \begin{array}{c} I\\ I\\ \begin{array}{c} \cdots \\ I \end{array} \end{array} & \begin{array}{c} \begin{array}{c} -I\\ 0 \end{array}\\ \begin{array}{c} \cdots \\ 0 \end{array} \end{array} & \begin{array}{c} 0\\ -I\\ \begin{array}{c} \cdots \\ 0 \end{array} \end{array}\\ \; & \begin{array}{cc} \begin{array}{c} \cdots \\ \cdots \\ \begin{array}{c} \cdots \\ \cdots \end{array} \end{array} & \begin{array}{c} 0\\ 0\\ \begin{array}{c} \cdots \\ -I \end{array} \end{array} \end{array} \end{array}\right]. \end{array}$$

If *χ*^2^ is found to be significant, individual differences ${\hat{\beta }}_{j}-{\hat{\beta }}_{l}$ may be considered concerning their approximate standard errors to elucidate the nature of the lack of fit.

### 3.4. Ethical Considerations

Permission to conduct the study was obtained from the University of Limpopo Turfloop Research and Ethics Committee (TREC/21/2019[NEI]) and the Department of Roads and Transport in Limpopo Province.

## 4. Results

**Figure 1:**
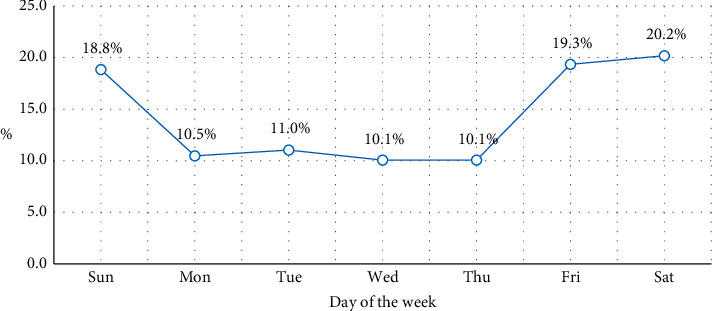
Weekly distribution characteristics of road accidents over a seven-year period.

Figure 1 illustrates a breakdown of RTAs by day of the week during a seven-year period in Limpopo Province of South Africa. The graph shows that weekends (Saturdays and Sundays) had the largest number of RTAs when compared to weekdays. The proportion of property damage, minor injuries, serious injuries, and mortality caused by an animal in a roadway was 82%, 83%, 79%, and 65%, respectively (shown in Table 2). The majority of fatal crashes occurred between 2:00 and 11:00 p.m., with 75.63% of property damage, 74.60% of minor injuries, 73.42% of serious injuries, and 55.41% of fatalities within those hours, shown in Table 2. The majority of traffic fatalities and injuries in this province happened on regional roads in Capricorn District. There was a significant linear trend observed for all the variables.

Table 3 shows the results of the ordered logit regression for associations between severity and related environmental factors in Limpopo Province, South Africa. Using a significance level of 0.05, the model findings show an association between environmental conditions, hour interval, road type, and region with the severity of injuries. Driving on roads with potholes, the odds of being more likely severe is 2.59 times higher than driving on animal-infested roadway, holding constant all other variables. The odds of being less severe is 0.67 lower than driving between 2:00 pm and 11:00 pm as compared to driving between 12:00 pm and 5:00 am. Driving on a provincial road, the odds are 0.30 less severe as compared to driving on districts roads holding all other variables constant. Driving in Mopani, Sekhukhune, Vhembe, and Waterberg districts is more likely severe than driving in Capricorn district.

The OLR model is based on the assumption that each independent variable has the same influence at each cumulative split of the ordered dependent variable. To check the model's adequacy and proportional odds assumption, the Hosmer-Lemeshow goodness-of-fit and Brant tests were performed. The goodness-of-fit test revealed that the model was well-fitting (likelihood ratio statistic = 5.88, degree of freedom = 9, *P* value = 0.7514). Brant's conclusion shows that the data did not meet the parallel lines assumption (chi-square = 74.04, degree of freedom = 38, *P* value = 0.0004), indicating that fitting the OLR model to the data was unsuccessful (shown in Table 4). As a result, in order to accommodate the proportionality constraint, we explore fitting the GOLR model. The model-fitting results are shown in Table 5.

Table 5 shows the results of the GOLR model for associations between deaths and severe injuries and related environmental factors in Limpopo Province, South Africa. Using a significance level of 0.05, the model findings show an association between environmental conditions, hour interval, season, road type, and region with severity. Driving on a slippery road is less likely to result in death and severe injuries than driving on animal inroads, suggesting that vehicle crashes are less likely to result in death and severe injuries when driving on a slippery road than when driving on the animal inroads. The odds of a car crash due to a stationary or parked vehicle on the road are 0.31 and 0.41, suggesting that vehicle crashes are less likely to result in minor injuries and death, respectively, compared to the odds of vehicle crashes due to animals on the roads. Vehicle crashes on poor or defective road surfaces were more likely to result in death and serious injuries. Driving in rainy weather is less likely to result in death and serious injuries than vehicle crashes due to animals on the roads.

K-means clustering was used as a clustering method for splitting the time of day into a set of *k* groups. We have grouped the time of day into 3 groups (00:00 a.m.–05:00 a.m., 06:00 a.m.–01:00 p.m., and 02:00 p.m.–11:00 p.m.) using the unsupervised k-means algorithm. The study results showed that the odds of a vehicle crash during 06–13 and 14–23 hours are 1.52 and 2.78, respectively, suggesting that vehicle crashes are more likely to result in deaths and severe injuries compared to the odds of a vehicle crash during 00–05 hours. Vehicle crashes during spring are less likely to result in deaths and severe injuries than driving during the autumn season. This suggests that vehicle crashes are less likely to result in deaths and severe injuries when driving in the spring season than during autumn. Vehicle crashes during winter were less likely to result in deaths against nondeaths.

Vehicle crashes on national roads are less likely to result in deaths and serious injuries than on district roads. Driving on provincial roads plays an important role in distinguishing the severity of vehicle crashes (death, minor, and serious injuries) from vehicle crashes that did not result in property damage but do not play a significant role in distinguishing the property damage and injuries from vehicle crashes that resulted in deaths. Regional roads were less likely to result in deaths and serious injuries as compared to district roads. Vehicle crashes in Mopani and Waterberg districts were less likely to result in deaths and injuries than in Capricorn district, suggesting that vehicle crashes are less likely to result in deaths and injuries when driving in Mopani and Waterberg districts than in Capricorn district. Vehicle crashes in Waterberg district were less likely to result in deaths against nondeaths as compared to Capricorn district.

## 5. Discussion

A study by Malin et al. found an increase in accident risks for poor road weather conditions such as heavy rain and slippery road conditions [15, 24, 25]. This might be because driving in rainy weather affects the driver's sight, the vehicle's traction, and the risk of an accident increases [15, 25, 26]. This is similar to the findings of the current study, which discovered that slippery road and rainy weather conditions were associated with deaths and severe injuries. Driving in rainy weather makes it difficult for drivers to maintain control of their vehicles since the road becomes more slippery [23]. Even though the majority of accidents occur due to bad weather [24–26], this study found that rain, slippery road conditions, and a stationary or parked vehicle significantly reduce road injury severity. Previous research came to similar conclusions, indicating that rain, snowy or slippery roads, and congested roadways decreased severity [27, 28]. This might be due to the fact that rainy weather causes reduction in the daily traffic volume and driving speed [29]. Our study findings also revealed that a poor or defective road surface increases the risk of death and serious injuries. Similar to earlier research, it was discovered that poor pavement conditions were linked to proportionally more severe injuries and that very poor pavement conditions were associated with fewer severe crashes [30, 31].

Our study also revealed that the hours of 06 a.m. to 1 p.m. and 2 to 11 p.m. significantly increased the likelihood of serious injuries to death when compared to the hours of 12 p.m. to 5 a.m., which may be attributed to the fact that the majority of individuals are going to and from various workplaces and schools. According to a study conducted by Meng (2017) estimating crash severity on mountainous freeways in Chongqing, accidents occurring between 19 and 24 p.m. were found to be more severe because of the driver's visual response, psychological load, and the road environment. The study also found that the degree of mutual adaptation is more unfavourable to the driver [32]. It was also stated that the likelihood of major traffic accidents is higher in summer and autumn than in the spring and winter. However, similar to the study findings, the likelihood of road severity against property damage decreased in spring compared to autumn, and the chance of death against nondeath decreased in winter season as compared to autumn. Summer was not found to be significantly associated with the severity of injuries.

It was reported in previous studies that minor and serious accidents are more frequent in urban areas, whereas fatal accidents are more likely in rural areas [33, 34]. This study found that national and provincial roads significantly reduce injury severity as compared to district roads. National roads are routes connecting major cities; provincial roads connect smaller cities and towns to the national route network; regional roads connect smaller towns to the route network; and district roads are mostly found in the rural areas where they connect market centres to provincial roads. Furthermore, in this study, it was revealed that Sekhukhune and Waterberg districts were less severe compared to Capricorn district. This might be due to the fact that Capricorn is a more developed district, and the capital city is situated in this region. Mopani and Vhembe were found not to be significantly associated with deaths and severe injuries.

The findings further showed that most road accidents in the province occurred during the weekend. This might be due to the increased congestion and traffic volume as a result of weekend travel. Contrary to this finding, Sangkharat et al. found a smaller number of road accidents on weekends compared to weekdays [35]. The disparity in the results might be attributed to weekend activities and traffic flow between these two countries (Thailand and South Africa). There was a significant linear trend for all the variables, suggesting that road accidents due to environmental factors vary according to injury severity. This is similar to findings in previous studies in which it was observed that rain, snowy or slippery roads, and busy highways reduce the severity [27, 28, 36].

Considering several strengths of the study, including the large sample size, limitations should be noted. The study used a secondary dataset collected and recorded by the Limpopo Provincial Department of Roads and Transport. It is noted that some important third variables were not available in the dataset, such as demographics (age, sex, race, and ethnicity), and road conditions such as road segment length, width, and number of ramps and bridges; therefore, when the omitted variables are significant covariates for injury severity, this might result in residual confounding. Moreover, another limitation comes from considering severe crashes from only one province.

## 6. Conclusions

The main purpose of this study was to determine the influence of environmental factors on injury severity and to identify whether their impact on injury severity levels varies. It was found in the study that the underlying assumption of the OLR model was violated to the extent that the relationship between each pair of outcome groups (property damage, minor, serious, and death) is not the same. The GOLR model seemed to be an effective predicting model that can be adapted to determine the influence of environmental factors on injury severity. The results of the present study suggest that environmental factors such as slippery road conditions, rainy weather, and spring season lower the likelihood of severe crash occurrence. On the other hand, poor or defective road surfaces, time intervals (6 a.m. to 11 p.m.), and provincial roads have a higher likelihood of severe crash occurrence. Finally, it may be concluded that frequent road maintenance on provincial roads is required.

## Figures and Tables

**Table 1:** Data categories and variables used in the study.

Variables	Description	Values
Severity level	Injury severity	1 = property damage; 2 = minor injury; 3 = serious injury; 4 = deaths
Day of week	One of the seven days in a week	1 = Monday 2 = Tuesday 3 = Wednesday 4 = Thursday 5 = Friday 6 = Saturday 7 = Sunday
District	Municipal district	1 = Capricorn; 2 = Vhembe; 3 = Mopani; 4 = Greater Sekhukhune; 5 = Waterberg
Season	Season	1 = autumn; 2 = spring; 3 = summer; 4 = winter
Road type	Categorized road type	1 = regional; 2 = district; 3 = national; 4 = others
Time	Categorized time intervals	1 = 00:00–05:59; 2 = 00:06–13:59; 3 = 14:00–23:59

**Table 2:** Frequency analysis of road accidents characteristics by property damage, death, and severe injuries.

Response	Property damage	Minor injury	Serious injury	Deaths	*P* trend
Environmental condition					<0.001
Animal in roadway	484 (81.90%)	366 (82.99%)	249 (78.80%)	48 (64.86%)	
Deposit on road	8 (1.35%)	5 (1.13%)	6 (1.90%)	4 (5.41%)	
Poor or defective road surface	21 (3.55%)	18 (4.08%)	10 (3.16)	2 (2.70%)	
Potholes	4 (0.68%)	9 (2.04%)	9 (2.85%)	3 (4.05%)	
Rain	48 (8.12%)	27 (6.12%)	23 (7.28%)	6 (8.11%)	
Slippery road	12 (2.03%)	12 (2.72%)	8 (2.53%)	3 (4.05%)	
Stationary or parked vehicle	14 (2.37%)	4 (0.91%)	11 (3.48%)	8 (10.81%)	
Hour interval					0.003
00–05 hours	65 (11.00%)	50 (11.34%)	50 (15.82%)	16 (21.62%)	
06–13 hours	79 (13.37%)	62 (14.06%)	34 (10.76%)	17 (22.97%)	
14–23 hours	447 (75.63%)	329 (74.60%)	232 (73.42%)	41 (55.41%)	
Season					<0.001
Autumn	177 (29.95%)	138 (31.29%)	100 (31.65%)	16 (21.62%)	
Spring	162 (27.41%)	135 (30.61%)	83 (26.27%)	19 (25.68%)	
Summer	114 (19.29%)	84 (19.05%)	73 (23.10%)	17 (22.97%)	
Winter	138 (23.35%)	84 (19.05%)	60 (18.99%)	22 (29.73%)	
Road type					<0.001
District	44 (7.45%)	41 (9.30%)	29 (9.18%)	6 (8.11%)	
National	73 (12.35%)	53 (12.02%)	51 (16.14%)	17 (22.97%)	
Others	79 (13.37%)	54 (12.24%)	31 (9.81%)	11 (14.86%)	
Provincial	14 (2.37%)	8 (1.81%)	1 (0.32%)	1 (1.35%)	
Regional	381 (64.47%)	285 (64.63%)	204 (64.56%)	39 (52.70%)	
Regional distribution					<0.001
Capricorn district	311 (52.62%)	195 (44.22%)	125 (39.56%)	15 (20.27%)	
Mopani district	129 (21.83%)	127 (28.80%)	84 (26.58%)	15 (20.27%)	
Sekhukhune district	23 (3.89%)	23 (5.22%)	23 (7.28%)	14 (18.92%)	
Vhembe district	52 (8.80%)	31 (7.03%)	31 (9.81%)	12 (16.22%)	
Waterberg district	76 (12.86%)	65 (14.74%)	53 (16.77%)	18 (24.32%)	

**Table 3:** The ordered logit regression for associations between severity and related environmental factors in Limpopo Province, South Africa.

Variable	Response	Odds (95% CI)	*t*-value	*P* value
Intercept	Property damage	Reference		
	Minor injury	0.7204 (0.2237; 1.2170)	−1.2938	0.1957
	Serious injury	2.8134 (2.3142; 3.3126)	4.0612	<0.0001^*∗*^
	Deaths	20.547 (20.006; 21.0876)	10.9534	<0.0001^*∗*^
Environmental condition	Animal in roadway	Reference		
	Deposit on road	1.4450 (0.6345; 3.2853)	0.8824	0.3775
	Poor or defective road surface	0.8679 (0.5089; 1.4664)	−0.5261	0.5987
	Potholes	2.5884 (1.2672; 5.3239)	2.6083	0.0090^*∗*^
	Rain	0.7898 (0.5252; 1.1821)	−1.1414	0.2536
	Slippery road	1.2668 (0.6690; 2.3845)	0.7323	0.4639
	Stationary or parked vehicle	1.9667 (0.9932; 3.8907)	1.9473	0.0514
Hour interval	00–05 hours	Reference		
	06–13 hours	0.6872 (0.4635; 1.0178)	−1.8698	0.0615
	14–23 hours	0.6737 (0.4999; 0.9084)	−2.5936	0.0094^*∗*^
Season	Autumn	Reference		
	Spring	1.0267 (0.7956; 1.3250)	0.2029	0.8391
	Summer	1.1466 (0.8649; 1.5197)	0.9522	0.3409
	Winter	0.9667 (0.7311; 1.2774)	−0.2375	0.8122
Road type	District	Reference		
	National	1.2356 (0.7690; 1.9885)	0.8734	0.3823
	Others	0.9559 (0.6049; 1.5123)	−0.1929	0.8470
	Provincial	0.3038 (0.1215; 0.7226)	−2.6386	0.0083^*∗*^
	Regional	1.0324 (0.7080; 1.5099)	0.1655	0.8684
Regional distribution	Capricorn district	Reference		
	Mopani district	1.5442 (1.2080; 1.9744)	3.4685	<0.0001^*∗*^
	Sekhukhune district	2.9979 (1.9280; 4.6739)	4.8665	<0.0001^*∗*^
	Vhembe district	2.0304 (1.3236; 3.1152)	3.2472	0.0017^*∗*^
	Waterberg district	1.6434 (1.2160; 2.2208)	3.2356	0.0012^*∗*^

^*∗*^ *P* value: significant at 0.05.

**Table 4:** The model goodness of fit using Brant test.

Variable	Response	Chi-square	Degree of freedom	*P* value
Environmental condition	Deposit on road	2.81	2	0.2448
	Poor or defective road surface	0.48	2	0.7853
	Potholes	0.40	2	0.8169
	Rain	1.76	2	0.4153
	Slippery road	0.07	2	0.9633
	Stationary or parked vehicle	13.82	2	0.0010^*∗*^
Hour interval	06–13 hours	2.87	2	0.2383
	14–23 hours	3.26	2	0.1964
Season	Spring	2.24	2	0.3263
	Summer	1.23	2	0.5410
	Winter	7.88	2	0.0194^*∗*^
Road type	National	1.35	2	0.5102
	Others	3.82	2	0.1483
	Provincial	3.66	2	0.1600
	Regional	1.05	2	0.5904
Regional distribution	Mopani district	1.03	2	0.5972
	Sekhukhune district	9.00	2	0.0111^*∗*^
	Vhembe district	10.56	2	0.0051^*∗*^
	Waterberg district	3.74	2	0.1540
Overall		74.04	38	0.0004^*∗*^

*P* value: significant at 0.05.

**Table 5:** The generalized ordered logit regression for associations between severity and related environmental factors in Limpopo Province, South Africa.

Variable	Response	Model 1	Model 2	Model 3
Intercept	Intercept	23.9693 (0.3775)	20.6316 (0.5987)	14.4687 (0.0090)^*∗*^
Environmental condition	Animal in roadway	Reference		
	Deposit on road	0.3857 (0.2536)	0.2575 (0.4639)	0.4376 (0.0514)
	Poor or defective road surface	1.6837 (0.6150)	1.7883 (0.0093)^*∗*^	1.5644 (0.8391)
	Potholes	0.1393 (0.3409)	0.3673 (0.8122)	0.5617 (0.3823)
	Rain	1.0803 (0.8470)	0.7133 (0.0083)^*∗*^	0.8623 (0.8684)
	Slippery road	0.5801 (<0.0001)^*∗*^	0.7383 (<0.0001)^*∗*^	0.7683 (0.0012)^*∗*^
	Stationary or parked vehicle	0.3121 (0.0012)^*∗*^	0.1161 (0.1957)	0.4131 (<0.0001)^*∗*^
Hour interval	00–05 hours	Reference		
	06–13 hours	1.5223 (<0.0001)^*∗*^	1.5757 (0.3775)	0.7782 (0.5987)
	14–23 hours	2.7720 (0.0090)^*∗*^	2.5924 (0.2536)	1.8745 (0.4639)
Season	Autumn	Reference		
	Spring	0.6137 (0.0514)^*∗*^	0.6509 (0.0615)	0.5641 (0.0094)^*∗*^
	Summer	0.6150 (0.8391)	0.5985 (0.3409)	0.7125 (0.8122)
	Winter	0.4669 (0.3823)	0.3731 (0.8470)	0.3706 (0.0083)^*∗*^
Road type	District	Reference		
	National	0.4982 (0.8684)	0.4421 (<0.0001)^*∗*^	0.6134 (<0.0001)^*∗*^
	Others	0.4571 (0.0011)^*∗*^	0.3405 (0.0012)^*∗*^	0.3379 (0.1957)
	Provincial	2.9075 (<0.0001)^*∗*^	2.1828 (<0.0001)^*∗*^	0.2473 (0.3775)
	Regional	0.8055 (0.5987)	0.6543 (0.0090)^*∗*^	0.8023 (0.2536)
Regional distribution	Capricorn district	Reference		
	Mopani district	0.3991 (0.4639)	0.6121 (0.0514)	0.6682 (0.0615)
	Sekhukhune district	0.0764 (0.0094)^*∗*^	0.1193 (0.8391)	0.1947 (0.3409)
	Vhembe district	0.1598 (0.8122)	0.1398 (0.3823)	0.3247 (0.8470)
	Waterberg district	0.2542 (0.0083)^*∗*^	0.3471 (0.8684)	0.3981 (<0.0001)^*∗*^

^*∗*^ *P* value: significant at 0.05; Model 1: injury severity vs. property damage; Model 2: death and serious injury vs. property damage and minor injury; Model 3: death vs. serious injury, minor injury, and property damage.

## Data Availability

The data supporting the findings of the article are available from the corresponding author upon reasonable request.
